# Adipokines and Arterial Stiffness in Obesity

**DOI:** 10.3390/medicina57070653

**Published:** 2021-06-25

**Authors:** Ioana Para, Adriana Albu, Mihai D. Porojan

**Affiliations:** 1 4th Department of Internal Medicine, University of Medicine and Pharmacy “Iuliu Hatieganu”, 400012 Cluj-Napoca, Romania; ioana.para@yahoo.com; 2 2nd Department of Internal Medicine, University of Medicine and Pharmacy “Iuliu Hatieganu”, 400012 Cluj-Napoca, Romania; porojan78@yahoo.com

**Keywords:** adipokines, arterial stiffness, cardiovascular risk

## Abstract

Adipokines are active molecules with pleiotropic effects produced by adipose tissue and involved in obesity-related metabolic and cardiovascular diseases. Arterial stiffness, which is a consequence of arteriosclerosis, has been shown to be an independent predictor of cardiovascular morbidity and mortality. The pathogenesis of arterial stiffness is complex but incompletely understood. Adipokines dysregulation may induce, by various mechanisms, vascular inflammation, endothelial dysfunction, and vascular remodeling, leading to increased arterial stiffness. This article summarizes literature data regarding adipokine-related pathogenetic mechanisms involved in the development of arterial stiffness, particularly in obesity, as well as the results of clinical and epidemiological studies which investigated the relationship between adipokines and arterial stiffness.

## 1. Introduction

Overweight and obesity have reached pandemic proportions, leading to an increase in type 2 diabetes mellitus, metabolic syndrome, and systemic hypertension. All these diseases play an essential role in the vascular aging process and contribute to an increase in cardiovascular morbidity and mortality. Obesity, characterized by an increased body mass index (BMI) ≥30 kg/m^2^, is an independent predictor of cardiovascular disease, while weight loss is associated with a reduction in obesity-related cardiovascular risk factors [1]. In addition to the increased total fat mass, cardiovascular risk is influenced by the distribution of fat deposits. Thus, visceral adipose tissue, which surrounds inner organs, is more pathogenic than increased subcutaneous fat since visceral obesity is more closely related to cardiovascular and metabolic diseases [2].

Adipose tissue is currently classified into three main types: white adipose tissue, which stores energy in the form of triglycerols; brown adipose tissue, involved in thermogenesis; and beige or bright adipose tissue, which contains inducible brown cells and has important thermogenetic roles. White adipose tissue is found in gluteofemoral, subcutaneous and visceral adipose tissue which surrounds various internal organs including the heart.

Over the last two decades, a lot of experimental and clinical studies have highlighted the important endocrine, autocrine and paracrine functions of the adipose tissue, particularly white adipose tissue, which produces physiologically active substances called adipokines. Adipose tissue-derived adipokines are involved in various metabolic and immune processes and contribute to maintaining body homeostasis [3]. Obesity is characterized by the hypertrophy and/or hyperplasia of adipocytes, adipose tissue inflammation and altered adipokine secretion, which are responsible for the obesity-related low-grade inflammation [4].

While visceral and subcutaneous fat produces systemic effects by the release of adipokines and other active molecules, perivascular fat, which is in direct contact with blood vessels, was found to have strong paracrine effects influencing the cardiovascular system independently of general obesity [5].

Arterial stiffness is the result of several important morphological modifications of the arterial wall, including increased collagen deposition, reduction in and fragmentation of elastin fibers, irreversible cross-links between advanced glycation end products and collagen, vascular smooth muscle cells (VSMCs) hypertrophy, and matrix calcification [6]. Aging is the most common condition associated with arterial stiffness [7]. The gold standard measure of great artery stiffness is aortic pulse wave velocity (PWV), which has an independent predictive value for all-cause and cardiovascular mortality [8]. However, other measures of arterial stiffness such as cardio–ankle vascular index (CAVI) and brachial–ankle PWV have proved to be important cardiovascular predictors. Moreover, carotid stiffness is associated with an increased risk of cerebral vascular complications [9].

Many epidemiological and clinical studies evaluating arterial distensibility in obese subjects have shown that obesity, particularly in its abdominal form, is associated with increased arterial stiffness [10,11,12,13]. Weight loss, induced by diet and lifestyle interventions, improved arterial stiffness [14]. In an experimental high-fat, high-sucrose-fed mouse model, obesity developed before or concomitantly with arterial stiffness, suggesting that arterial modifications are a consequence of obesity. Moreover, a normal caloric diet induced weight loss and decreased arterial stiffness [15].

The pathogenesis of arterial stiffness in obesity is not completely elucidated. Insulin resistance, systemic inflammation, oxidative stress, renin–angiotensin–aldosterone system (RAAS) activation and increased vascular tone due to the activation of the sympathetic nervous system are some important mechanisms linking obesity to vascular disease. Data from experimental and human studies support the involvement of adipokines in the complex mechanisms that induce obesity-related arterial stiffness [12,16].

**Figure 1:**
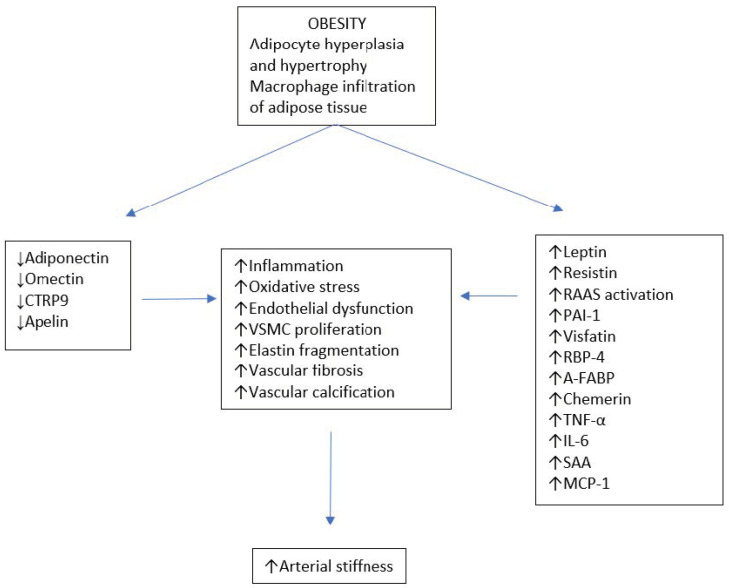
Obesity-induced adipokines’ modifications and arterial stiffness. Abbreviations: VSMC, vascular smooth muscle cell; RAAS, renin–angiotensin–aldosterone system; PAI-1, plasminogen activator inhibitor-1; RBP-4, retinol binding protein-4; A-FABP, adipocyte-fatty acid binding protein; TNF-α, tumor necrosis factor-α; IL-6, interleukin-6; SAA, serum amyloid A; MCP-1 monocyte chemoattractant protein-1.

Adipokines are cytokines produced by various types of cells, including adipocytes, macrophages and lymphocytes, particularly in white adipose tissue. According to their relationship with systemic inflammatory response, adipokines have anti-inflammatory or pro-inflammatory effects. The great majority of adipokines are pro-inflammatory, including interleukin-6 (IL-6), tumor necrosis factor-alpha (TNF-α), adipocyte fatty acid-binding protein (A-FABP), plasminogen activator inhibitor-1 (PAI-1), resistin, leptin, and monocyte chemoattractant protein-1 (MCP-1). They are related to obesity metabolic and vascular complications. A small number are anti-inflammatory, protective adipokines, such as adiponectin omentin, C1q/TNF-related protein-9 (CTRP9) and apelin. In obesity, the increase in white adipose tissue from visceral deposits shifts the balance in favor of pro-inflammatory molecules [17], activating mechanisms that may subsequently induce arterial stiffness (Figure 1).

This review summarizes the literature data regarding the adipokine-related pathogenetic mechanisms involved in the development of arterial stiffness, particularly in obesity, as well as the results of clinical and epidemiological studies investigating the relationship between adipokines and arterial stiffness.

## 2. Anti-Inflammatory Cytokines and Arterial Stiffness

### 2.1. Adiponectin

Adiponectin is mainly expressed in adipose tissue [18]. It is synthesized by both brown and white adipose tissue, but it is higher in subcutaneous white adipose tissue compared to visceral tissue [19]. Plasma adiponectin is negatively correlated with adipose tissue mass. Its levels are decreased in obesity and metabolic syndrome but increased with weight loss [20,21]. Furthermore, reduced adiponectin is associated with pathological states related to obesity. It has been suggested that reduced plasma adiponectin levels may have an essential role in the development of insulin resistance, type 2 diabetes, and metabolic syndrome [22]. Several forms of adiponectin can be identified: the trimer and hexamer (lower molecular weight forms), and the high molecular weight form (HMW). Among these forms, HMW adiponectin is the most active [23]. Adiponectin exerts its actions through the activation of two types of receptors, adiponectin receptor 1 (AdipoR1) and adiponectin receptor 2 (AdipoR2). AdipoR1 mediates the activation of AMP-activated protein kinase (AMPK) and is predominantly found in muscle and endothelial cells. AdipoR2 is mainly expressed in the liver and is linked to the activation of peroxisome proliferator-activated receptor alpha (PPAR-α) [24,25]. Adiponectin plays important roles in regulating lipid and glucose metabolism and in reducing inflammation and oxidative stress [25,26,27]. A decreased expression of adiponectin receptors has been found in experimentally induced obesity and insulin resistance [28].

**Figure 2:**
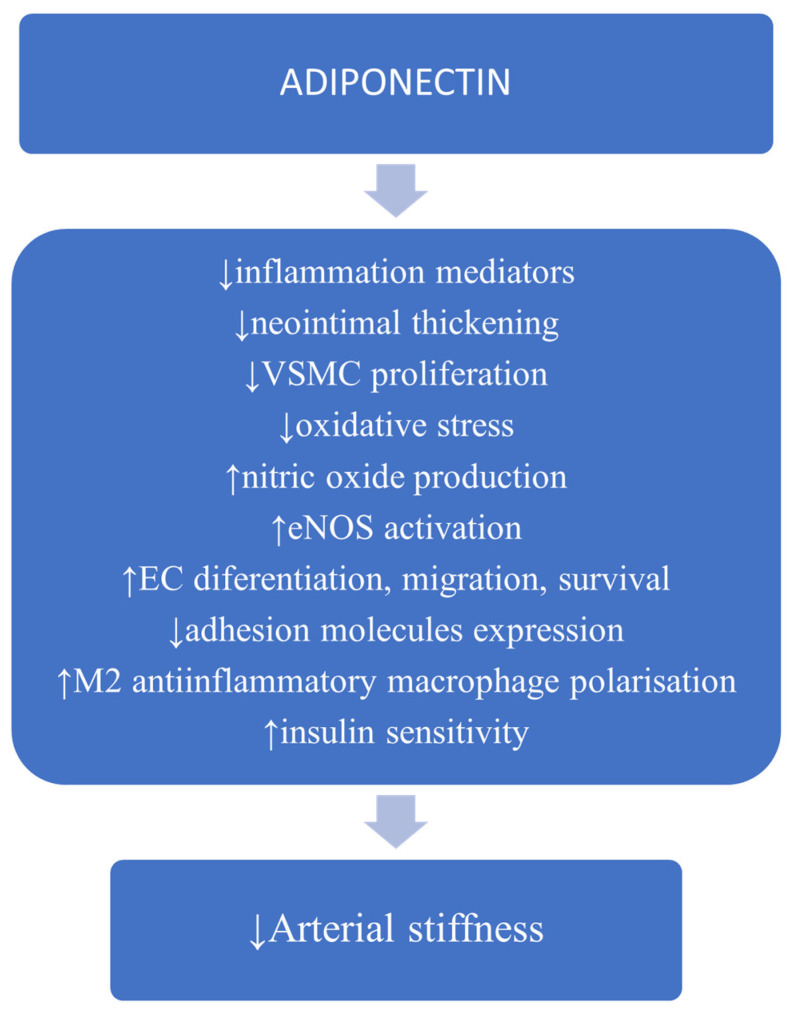
Possible mechanisms involved in adiponectin protection against the development of arterial stiffness (information based on cited articles). Abbreviations: VSMC, vascular smooth muscle cell; eNOS, endothelial nitric oxide synthase; EC, endothelial cell.

At the level of the vascular wall, adiponectin inhibits endothelial nuclear transcription factor NF-kB signaling, suppressing inflammatory cytokine production [29], reduces the expression of endothelial adhesion molecules [30], inhibits vascular smooth muscle cell (VSMC) proliferation and migration [31]. Moreover, adiponectin promotes the transformation of the M1 macrophage pro-inflammatory profile to the M2 anti-inflammatory type, thus reducing inflammation [32], diminishes the apoptosis of endothelial cells and stimulates their differentiation and migration [31]. Adiponectin increases nitric oxide (NO) availability with favorable effects on endothelial function [32]. Hypoadiponectinemia has been associated with endothelial dysfunction in patients with hypertension and diabetes mellitus [33,34] while increased levels of adiponectin may ameliorate endothelial function [35]. All these vascular effects can favorably impact arterial stiffness (Figure 2).

The relationship between adiponectin and arterial stiffness has been evaluated in clinical and epidemiological studies. The correlation with various markers of obesity and with fat mass distribution has also been investigated.

In extremely obese subjects (BMI > 40 kg/m^2^), carotid distensibility decreased and intima–media thickness (IMT) increased when compared to a control group with a BMI of 18.5 to 30.0 kg/m^2^. Obese subjects had suppressed adiponectin and an increased leptin-associated inflammatory profile expressed by higher C-reactive protein (CRP) and low interleukin 10. Adiponectin positively correlated with carotid distensibility. This association was independent of classical cardiovascular risk factors (including age, BMI and systolic blood pressure) and inflammation markers, suggesting that adiponectin may be a valid marker of carotid stiffness in these subjects [36].

In never-treated hypertensive subjects, adiponectin was negatively associated with aortic PWV, independently of classical cardiovascular risk factors, including BMI and systemic inflammation. The mechanism involved in this association could not be identified but an involvement of insulin resistance was speculated [37].

The relationship of adiponectin with fat mass distribution and with local carotid, femoral and brachial stiffness was evaluated in a research sample that included 456 elderly participants (age ranging from 60 to 86 years), from the Hoorn study. Higher adiponectin was associated with reduced peripheral arterial stiffness at all three arterial sites, particularly in patients without cardiovascular disease and diabetes mellitus. There were no correlations with central arterial stiffness. Trunk fat associated with lower adiponectin levels while leg fat correlated with higher levels, which suggested that these fat depots differ in their adiponectin secretion rate. Nevertheless, the authors found that adiponectin only explained a small part of these associations and suggested that other factors such as insulin, glucose levels, inflammatory markers or other adipokines may influence the association between fat mass distribution and arterial stiffness [38]. Moreover, in this study, central stiffness, which is more closely related to cardiovascular risk than peripheral stiffness [7,8], did not correlate with adiponectin levels.

In patients with prior cardiovascular disease [39,40,41] a positive association has been reported between adiponectin and measures of vascular stiffness. A positive correlation between adiponectin and arterial stiffness has also been reported in chronic kidney disease patients [42]. It has been suggested that increased systemic inflammation in these conditions may have influenced the relationship between adiponectin and vascular parameters. Another hypothesis could be that adiponectin synthesis might be stimulated as a compensatory reaction to protect vessels. Studies in patients with diabetes mellitus reported conflicting results regarding the relationship between adiponectin and arterial stiffness [43,44,45], suggesting that adiponectin’s role in diabetic vascular disease may be masked or influenced by other factors.

High molecular weight (HMV) adiponectin, the most active form of adiponectin, has also been studied in relation to arterial stiffness. In a community-dwelling persons study, age-adjusted heart brachial and heart ankle PWV progressively decreased with increased serum HMW adiponectin. Decreased serum HMW adiponectin levels were significantly and independently associated with increased mean PWV, suggesting a protective vascular effect of HMW adiponectin [46].

In prospective studies, low adiponectin levels at baseline correlated with increased arterial stiffness at follow-up. In 142 healthy non-diabetic postmenopausal women, between 40 and 70 years of age, with carotid IMT more than 1 mm at least in one of the three measured segments (the distal 10 mm of the common carotid artery, the carotid bulb, and the 10 mm of the internal carotid artery), low adiponectin was associated with 3-fold increased risk of carotid IMT progression and a 1.7-fold increased risk of arterial stiffness progression after 1-year follow-up. This association was not influenced by traditional cardiovascular risk factors. Adiponectin correlated with CRP but not with intercellular adhesion molecule-1 (ICAM-1) and vascular cell adhesion molecule-1 (VCAM-1), suggesting that adiponectin vascular effects may not be explained by adhesion molecules [47]. Another prospective study, which included 3769 participants, evaluated the relationship between classical cardiovascular risk factors and inflammatory markers and adiponectin measured at baseline, and carotid–femoral PWV determined 16 years later. Central obesity and low-grade inflammation (IL-6, interleukin1-receptor antagonist, CRP, and fibrinogen) were strong predictors of arterial stiffness in both sexes, while low adiponectin predicted aortic stiffness in women but not in men, suggesting a sex differences in the long-term determinants of vascular stiffness [48]. However, in a study that included 240 middle-aged men free of cardiovascular disease at baseline, low baseline adiponectin correlated with greater increase in carotid–femoral PWV after 4.6 years of follow-up [49]. In treated hypertensive patients, low baseline adiponectin levels predicted increased arterial stiffness measured as cardiac–femoral PWV, after 2 years of follow-up [50].

The results of these studies suggested that low adiponectin levels may be not only a marker of vascular disease but also a mediator. However, adiponectin may have different effects depending on the studied population.

### 2.2. Omentin-1

Omentin-1 is mainly secreted by stromal vascular cells of human visceral fat. Obesity is accompanied by low levels of omentin [51]. It has been shown that omentin reduces endothelial TNF-α and TNF-α-induced expression ICAM-1 and VCAM-1, by the nuclear factor kappa B (NF-κB) inhibition, in cultured endothelial cells [52]. Experimentally, omentin-1 transgenic mice (ApoE-KO/OMT-Tg mice) showed to have reduced aortic macrophage infiltration and expression of proinflammatory cytokines (TNF-α, IL-6 and MCP-1) compared to ApoE-KO mice [53]. Omentin-1 also has other favorable effects such as the stimulation of endothelial NO synthase-dependent vasodilatation in aortic rings, reduces vascular inflammation, neointimal intimal thickening, and vascular cell proliferation in injured vessels [31,54]. All these effects support a favorable vascular action of omentin, which may be associated with reduced arterial stiffness.

In a study which included type 2 diabetic patients, omentin-1 was decreased compared to normal glycemic patients and correlated with brachial ankle PWV and carotid plaques after multiple adjustments for cardiovascular risk factors and for medication history [55]. A prospective investigation including patients with type 2 diabetes mellitus found that baseline omentin positively correlated with changes in arterial stiffness within one year, even after adjusting for classical cardiovascular risk factors and CRP [56].

### 2.3. CTRP9

The CTRP family comprises 15 members which are considered adiponectin paralogs as they share structural similarities with adiponectin. Among all of these, CTRP9 presents the greatest amino acid structural similarities with adiponectin [31]. Experimental research has reported favorable cardiovascular effects of this adipokine. CTRP9 acts via Adipo R1 receptors stimulating NO production in endothelial cells and endothelial dependent vasodilatation CTRP9 reduces endothelial inflammation and oxidative damage using the AdipoR1/AMPK activation pathway [57]. It also reduces VSMCs proliferation [58].

In human studies, CTRP9 has been associated with good glycemic control and an absence of metabolic syndrome [59]. Its values were decreased in patients with coronary artery disease compared to healthy controls [60]. However, increased levels of CTRP9 have been positively associated with arterial stiffness [61] and carotid intima–media thickness [62] in diabetic patients, suggesting a compensatory vascular protective increase.

### 2.4. Apelin

Apelin is produced in many tissues, including fat tissue, brain, heart, lungs and kidneys. It has favorable metabolic effects reducing insulin resistance with possible anti-obesity and anti-diabetic properties [26]. Apelin binds to its vascular APJ receptors inducing endothelium-dependent vasodilation, predominantly mediated through increased NO production [26,63]. This effect may reduce arterial stiffness and systemic hypertension [64]. Regular aerobic training in middle-aged and elderly subjects reduced arterial stiffness and concomitantly increased circulating apelin and NO. Apelin may thus contribute to a reduction in arterial stiffness by increasing NO during aerobic exercise [65].

## 3. Proinflammatory Cytokines and Arterial Stiffness

### 3.1. Leptin

Leptin is mainly produced by the differentiated adipocytes of the white subcutaneous adipose tissue. Leptin has an important role in regulating food intake and energy expenditure in the central nervous system. It reduces appetite and increases energy expenditure. Nevertheless, in obesity, increased fat mass is accompanied by hyperleptimenia, suggesting a state of leptin resistance [26].

The vascular effects of leptin are mediated via specific vascular receptors located in endothelial cells and in VSMCs [66]. The direct action of leptin on the vascular wall is supported by an experimental study which found that ob/ob mice, which lack leptin, became hyperphagic and obese but did not develop atherosclerosis [67].

However, leptin may have opposing vascular and metabolic actions. Experimental studies have shown that leptin favors endothelium-dependent NO-mediated vasorelaxation, corrects impaired antioxidant defense in ob/ob mice and protects macrophages from cholesterol overload. Leptin has been shown to have anti-insulin-resistant properties [68,69]. In contrast, in obesity, chronic hyperleptinemia may produce, by various mechanisms, deleterious cardiovascular effects and insulin resistance [70]. Leptin-induced RAAS activation and entothelin-1 synthesis may contribute to endothelial dysfunction and obesity-associated arterial hypertension [69,71]. Leptin may activate the renal sympathetic nervous system causing increased renal sodium retention and subsequently, increased blood pressure [72].

**Figure 3:**
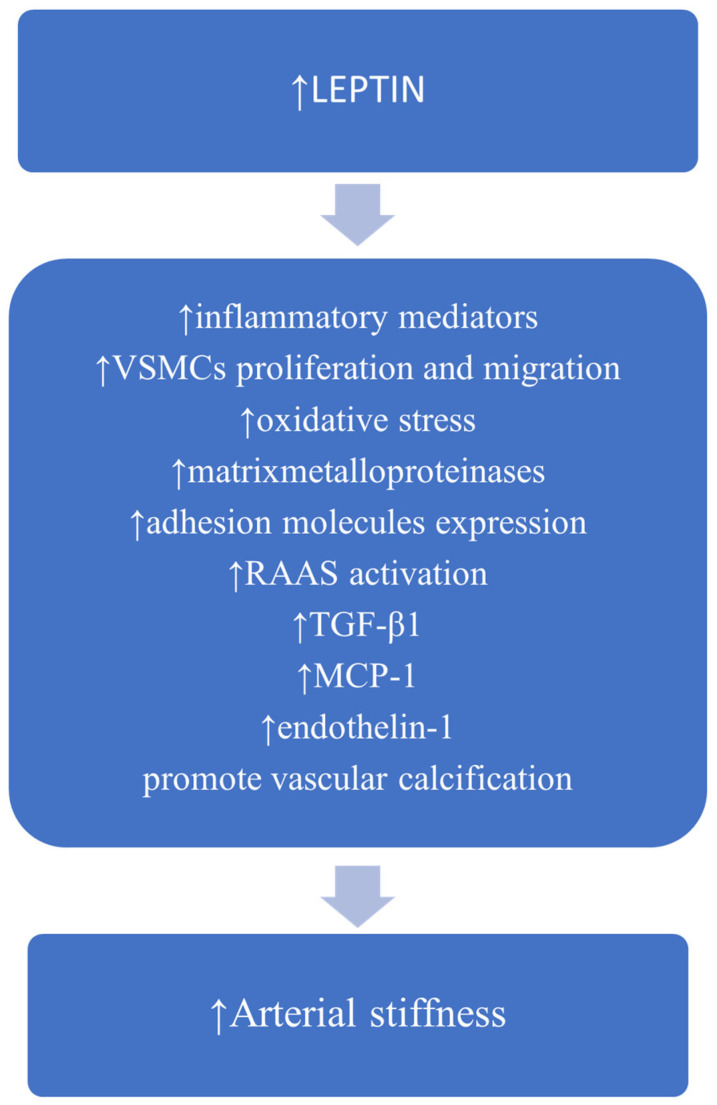
Possible mechanisms linking leptin to increased arterial stiffness (information based on cited articles). Abbreviations: VSMC, vascular smooth muscle cell; RAAS, renin–angiotensin–aldosterone system; TGF-β1, transforming growth factor- β1; MCP-1, monocyte chemoattractant protein-1.

Obesity-induced hyperleptinemia is involved in vascular inflammation. It stimulates the expression of inflammatory cytokines, such as TNF-α, interleukine-2, and IL-6 by monocytes [73] and lymphocyte T helper 1 proinflammatory cytokine production [74]. In aortic endothelial cells, leptin induces MCP-1 synthesis by increasing fatty acid oxidation via protein kinase A [75]. Leptin may stimulate VSMC proliferation and migration, enhance the expression of matrix metalloproteinases, adhesion molecules and transforming growth factor-β1 (TGF-β1) [76,77]. Leptin increases oxidative stress by multiple mechanisms including the activation of protein kinase A, which increases fatty acid oxidation in endothelial cells [75] and the decrease in paraoxonase 1 antioxidant activity [78]. Moreover, leptin may promote osteoblastic differentiation and the calcification of vascular cells [79]. Possible mechanisms linking leptin to arterial stiffness are depicted in Figure 3.

Leptin has been studied in relation to arterial stiffness, alone or concomitantly with other adipokines in clinical and epidemiological studies.

In 294 healthy adolescents, leptin positively correlated with carotid–femoral PWV, independently of body fat mass, blood pressure and CRP. It has been suggested that the vascular effects of leptin may begin early in life, even in non-obese but slightly overweight young subjects, and subsequently, the prolonged action of leptin on the vasculature may increase the risk of adult cardiovascular disease onset [80]. This study supports a direct effect of leptin on the vascular wall, previously suggested by experimental findings [67].

A cross-sectional analysis from the Baltimore Longitudinal Study of Aging which included 749 patients (mean age 67 years) investigated adiponectin, leptin and resistin in relation with carotid–femoral PWV and abdominal obesity. This study indicated that increased leptin and resistin and low adiponectin independently correlated with increased arterial stiffness, however, the relationship between each adipokine and stiffness measure was not influenced by the magnitude of abdominal adiposity, suggesting that factors other than obesity may influence the relationship between adipokines and arterial stiffness. Among the three studied adipokines, only leptin explained, in part, the relationship between abdominal obesity and arterial stiffness, because the inclusion of leptin in the statistical model attenuated the relationship between obesity and vascular stiffness [81].

In healthy elderly men, leptin correlated with arterial stiffness and the association of increased leptin with low adiponectin levels had a synergistic effect on the development of subclinical vascular damage indicated by increased carotid–femoral PWV (>10 m/s) [82]. The proinflammatory effect of leptin seemed to be balanced by the adiponectin anti-inflammatory effect. Adiponectin may inhibit the remodeling of the vascular extracellular matrix promoted by leptin which induces vessel fibrosis and subsequently arterial stiffness [83]. The association of increased adiponectin and low leptin has been found in subjects with favorable metabolic profile [84] and increased life expectancy [85].

In a large population from the SardiNIA Study, the correlation between metabolic syndrome, aortic stiffness, and carotid IMT and a panel of adipokines including adiponectin, leptin, IL-6, and MCP-1 was investigated. Higher leptin levels were positively associated with increased aortic PWV, while low adiponectin was correlated with increased carotid intima–media thickness, after adjusting for age, sex and other classical cardiovascular risk factors. The association of increased inflammatory cytokines with metabolic syndrome showed to have a synergistic effect on arterial stiffness and thickness [86]. This study indicated that in subjects with metabolic abnormalities, the presence of an inflammatory adipokine profile may increase the risk of vascular alteration and impose more effective therapeutic interventions.

Increased leptin positively correlated with systolic blood pressure and pulse pressure and negatively with arterial compliance in obese/overweight hypertensive, compared to obese/overweight normotensive African women independently of obesity, insulin resistance, and age [87]. These results suggest a possible involvement of leptin in arterial stiffness development in hypertensive obese African women.

Positive correlations between leptin and increased carotid–femoral PWV have also been reported in patients with hypertension [88], coronary artery disease [89], chronic kidney disease patients in predialytic stages [90] and also in hemodialysis patients [91]. In patients with type 2 diabetes mellitus, leptin independently correlated with brachial–ankle PWV [92].

However, in patients with non-alcoholic fatty liver disease, the leptin/adiponectin ratio did not correlate with brachial–ankle PWV, suggesting that other factors may contribute to vascular alterations in these patients [93]. Moreover, in postmenopausal women followed up prospectively over one year, adiponectin but not leptin predicted the increase in carotid thickness and stiffness [47].

### 3.2. Resistin

The name “resistin” was established based on its action of increasing insulin resistance. In animal models, increased levels of resistin induced hyperglycemia associated with elevated hepatic glucose production while the reduction in resistin prevents obesity-related hyperglycemia due to increased insulin sensitivity [20]. In humans, several studies found an association between resistin and obesity, while weight loss was accompanied by a reduction in resistin level [94]. Other studies failed to demonstrate a positive relation between resistin levels and insulin resistance, and a clear role of resistin in mediating insulin resistance in obesity and diabetes cannot yet be supported [62,94]. However, a recent meta-analysis found that resistin levels are correlated with insulin resistance in obese and type 2 diabetes mellitus patients [95].

In human adipose tissue, resistin is mainly produced by mononuclear cells [96] and is involved in various mechanisms which may cause adverse vascular effects. Resistin showed to have proinflammatory effects by promoting the activation of NFκB transcription factor and stimulating the secretion of pro-inflammatory molecules, such as IL-6 and TNF-α [97,98,99]. Resistin stimulates the proliferation and migration of vascular endothelial and VSMCs [99,100], induces endothelial dysfunction and contributes to increased endothelial cell oxidative stress [26,62]. This increases endothelial secretion of endothelin-1, a potent vasoconstrictor [98] and the expression of endothelial adhesion molecules [101,102]. Vascular proinflammatory effects and the metabolic disturbances may negatively impact cardiovascular outcomes. Indeed, resistin has been associated with an increased risk of cardiovascular and all-cause mortality [103].

Positive correlations between resistin and arterial stiffness have been reported. In a population study which included 683 randomly selected subjects of African ancestry from Soweto, South Africa, who had never taken antihypertensive therapy, resistin positively correlated with aortic PWV independently of age, blood pressure, and systemic inflammation [104]. Higher resistin levels were associated with increased aortic stiffness in patients with coronary artery disease, independently of age, diabetes mellitus, waist circumference, and CRP [105].

### 3.3. Renin–Angiotensin–Aldosterone System

All RAAS components can be produced by the adipose tissue, particularly by visceral and perivascular adipose tissue. Angiotensinogen is the precursor of RAAS. Renin cleaves angiotensinogen to produce angiotensin I which, subsequently, is transformed by angiotensin converting enzyme into angiotensin II. Chronic exposure to increased aldosterone, caused by adipocyte production in obesity, induces vascular remodeling and fibrosis as well as endothelial dysfunction. In addition, vascular mineralocorticoid receptor activation in obesity contributes to impaired vascular relaxation and oxidative stress [106]. Increased vascular mineralocorticoid receptor activation activates TGF-β1 leading to vascular fibrosis. The administration of mineralocorticoid receptor antagonist spironolactone prevents the development of aortic stiffness in Western diet-induced arterial stiffness of obese female mice [107]. Aldosterone was shown to induce the stiffness of the endothelial cells cytoskeleton as well as VSMCs stiffness. It may also stimulate the expression of ICAM-1 [108]. Angiotensin II induces renal sodium reabsorption and vasoconstriction, and subsequently, increases blood pressure. Obese subjects have increased levels of adipose tissue-derived angiotensinogen which may contribute to obesity-related arterial hypertension [109]. Moreover, angiotensin II stimulates the release of leptin, PAI-1 [110], and inflammatory cytokines [111].

Weight loss, one month after bariatric surgery, in patients with severe obesity, significantly improved arterial stiffness. Changes in some components of RAAS were independent correlates for the final values of vascular parameters, suggesting the involvement of this system in obesity-associated arterial stiffness [112].

Favorable effects of the RAAS blockade on arterial stiffness have been reported in obese hypertensive patients. Blocking RAAS with angiotensin converting enzyme inhibitors or angiotensin receptor blockers showed to be effective in reducing arterial stiffness and vascular remodeling in hypertensive patients [113].

### 3.4. Plasminogen Activator Inhibitor-1

PAI-1 is an essential inhibitor of tissue plasminogen activator. Increased PAI-1 activity is associated with reduced fibrinolytic activity and thus with increased cardiovascular risk. Adipose tissue, particularly visceral fat, is an important source of PAI-1. In addition to its role in controlling fibrinolysis, PAI-1 is also involved in vascular remodeling. PAI-1 may modulate neointima formation, VSMCs proliferation and migration, and TGF-β1 activation [114]. Both PAI-1 and TGF-β1 contribute to vascular fibrosis and are increased in experimental and human models of aging [115].

In human studies, PAI-1 was associated with insulin resistance, metabolic syndrome, and atherosclerosis [116,117]. In untreated hypertensive subjects, PAI-1 positively correlated with markers of carotid stiffness, suggesting that decreased fibrinolytic activity may be involved in early vascular wall alteration [118]. Decreased carotid distensibility correlated with markers of insulin resistance, including PAI-1, in non-diabetic middle-aged women [119]. In a community-based sample from the Framingham Heart Study, PAI-1 positively correlated with mean arterial pressure, central pulse pressure, and forward pressure wave amplitude after adjustment for classical cardiovascular risk factors [120].

### 3.5. Visfatin

Visfatin (as its name suggests) is an adipokine mainly secreted by the visceral adipose fat. It has also been found in great quantities in the perivascular adipose tissue. Visfatin is also known as pre-B cell colony enhancing factor because it acts as a growth factor for early state B cells. In humans, the principal source of visfatin is represented by macrophages from the adipose tissue, which are stimulated by inflammatory signals [121]. Visfatin synthesis increases in obesity and stimulates the expression of inflammatory cytokines such as IL-6 and TNF-α, adhesion molecules, metalloproteinases (MMP-2 and MMP-9), VSMCs proliferation and migration, and collagen synthesis. All these effects may contribute to vascular remodeling [122]. However, its precise role in the development of atherosclerosis and arterial stiffness has not yet been clarified.

In humans, visfatin, expressed by periaortic and pericoronary adipose tissue, is strongly correlated with atherosclerosis [123] and with unstable carotid and coronary plaques [124]. Visfatin was inversely correlated with endothelial function [125] and was an independent predictor of increased carotid IMT [126]. Together with high-sensitivity CRP, visfatin was increased in patients with spinal cord injury compared to healthy controls and positively correlated with carotid stiffness [127]. However, in hemodialysis patients, visfatin correlated with high-sensitivity CRP but not with aortic PWV, suggesting that visfatin may reflect systemic inflammation rather than vascular wall changes [128].

### 3.6. Retinol Binding Protein (RBP)-4

RBP-4 is an adipokine mainly produced by the liver and visceral fat [129]. It increases in obesity and diabetes mellitus and may cause insulin resistance [130]. The Framingham study showed that RBP-4 was associated with insulin resistance and high blood pressure but not with body mass index [131]. However, RBP-4 proved to be more closely correlated with visceral than subcutaneous fat [132]. In obese individuals, RBP-4 was associated with an increased risk of cardiovascular morbidity [131].

Several mechanisms can explain the RBP-4 involvement in vascular pathology. RBP-4 may increase oxidative stress and stimulate vascular endothelium inflammation leading to endothelial dysfunction. Moreover, RBP-4 induces the macrophage secretion of inflammatory cytokines, including TNF-α, IL-6, IL-2, and MCP-1. It has been associated with a non-favorable lipid profile [133] which may also contribute to vascular alterations.

RBP-4 has been positively correlated with arterial stiffness measured as carotid–femoral PWV in healthy postmenopausal women [134] and in adolescents with family history of type 2 diabetes [135]. In a large study, which included subjects with various cardiometabolic risk factors, urinary RBP-4, which might be the consequence of increased circulating RBP-4, was positively associated with brachial–ankle PWV, insulin resistance, microalbuminuria, and inflammation [136].

### 3.7. Adipocyte Fatty Acid Binding Protein (A-FABP)

A-FABP is mainly produced by the adipose tissue and macrophages from the subcutaneous fat. It is involved in inflammatory and metabolic processes. Increased levels of A-FABP have been correlated with insulin resistance, vascular inflammation, endothelial dysfunction, and the development of atherosclerosis [137].

In clinical studies, A-FABP has been positively associated with obesity and metabolic syndrome [138]. Arterial stiffness parameters have been positively associated with A-FABP levels in various populations. In the large Framingham Third Generation cohort study, which included healthy young and middle-aged participants free of cardiovascular disease, A-FABP positively correlated with carotid–femoral PWV after adjustment for various confounders, including BMI [139]. In hypertensive patients, A-FABP predicted metabolic syndrome and arterial stiffness. Increased A-FABP values were positively associated with carotid–femoral PWV in patients with hypertension and metabolic syndrome and not in hypertensive patients without metabolic syndrome, emphasizing the possible role of this adipokine in obesity and metabolic syndrome-mediated vascular disease [140]. Recently, A-FABP was shown to be an independent predictor of carotid–femoral PWV in type 2 diabetes mellitus patients [141]. A previous study found that type 2 diabetes mellitus patients had increased levels of A-FABP and reduced levels of adiponectin. A-FABP positively correlated with aortic augmentation index, a marker of arterial stiffness and wave reflection [142]. In newly diagnosed hypertensive patients, circulating A-FABP positively correlated with BMI, homeostasis model assessment as a marker of insulin resistance, and systolic blood pressure. There was also a tendency for correlation between A-FABP and CAVI, a measure of central arterial stiffness. Treatment with Olmesartan for 6 months resulted in a significant reduction in A-FABP, CAVI, high-sensitivity CRP and blood pressure. The change in CAVI was correlated with the change in A-FBP in multiple regression analysis. It has been speculated that Olmesartan may reduce oxidative stress and inflammation with a favorable impact on arterial stiffness and A-FABP values [143]. In elderly patients, aged 65 or older, subjects with increased carotid–femoral PWV had increased levels of circulating A-FABP. Moreover, A-FABP was an independent predictor for the development of arterial stiffness [144]. Circulating A-FABP correlated with carotid–femoral PWV in patients on peritoneal dialysis [145] and with CAVI in kidney transplant patients [146] suggesting a large correlation between this adipokine and various arterial stiffness parameters, which could not only be explained by its involvement in insulin resistance and systemic inflammation.

### 3.8. Chemerin

Chemerin, an adipokine secreted by the white adipose tissue, is significantly higher in obese individuals [147]. Weight reduction was accompanied by a decrease in circulating chemerin [148]. Chemerin showed to participate in activation of NF-κB, suggesting a possible implication in obesity-related inflammatory reaction. Moreover, it acts as a chemoattractant for macrophages and dendritic cells, induces ICAM-1 and E-selectin expression [149] and may also enhance MMP-2 and MMP-9 expression in endothelial cells, contributing to extracellular matrix remodeling [150]. Chemerin is involved in the regulation of vascular tone inducing endothelial dysfunction, the contraction of VSMCs and enhancing sympathetic nerve function [151].

In clinical studies, a direct correlation between chemerin and various markers of arterial stiffness has been reported. In 120 healthy subjects, chemerin correlated with brachial–ankle PWV independently of classical cardiovascular risk factor, homeostasis model of insulin resistance, and high-sensitivity CRP [148]. An independent correlation between chemerin and brachial–ankle PWV was also reported in subjects with metabolic syndrome [152]. In newly diagnosed hypertensive subjects, chemerin positively correlated with brachial–ankle PWV and negatively with brachial flow-mediated dilation after adjustment for metabolic variables and inflammatory markers (high-sensitivity CRP, tumor necrosis factor-α, and interleukin-4) [153].

Local perivascular and epicardial chemerin expression may play a role in vascular pathology. Increased secretion of chemerin in perivascular and epicardial tissue has been associated with aortic and coronary atherosclerosis. Moreover, hypertensive subjects had increased chemerin secretion in periaortic adipose tissue and aortic VSMCs compared to normotensives, suggesting a possible role of the perivascular fat secretion of chemerin in blood pressure control [154,155].

Nevertheless, in patients with chronic kidney disease, chemerin was associated with low coronary calcium score despite positive correlation with renal function, cardiometabolic risk factors and inflammation. Chemerin was also correlated with two calcification inhibitors, fetuin-A and matrix Gla protein. In murine VSMCs, chemerin increased matrix Gla protein and reduced calcification. In contrast with previously mentioned studies, chemerin did not correlate with the augmentation index, a marker of arterial stiffness, which depends on vascular calcification as well as on vascular fibrosis and endothelial function [156]. All these results suggest a protective vascular function of chemerin in chronic kidney disease patients, although the pathogenic mechanisms of these effects are not yet clarified.

### 3.9. Tumor Necrosis Factor-α (TNF-α) and Interleukin 6 (IL-6)

TNF-α and IL-6 are two cytokines involved in systemic inflammatory syndrome.

TNF-α is mainly secreted by macrophages which infiltrate adipose tissue, while its adipocyte synthesis is reduced [157]. In obese subjects, there is an increased TNF-α production which plays an essential role in the development of insulin resistance and local adipose tissue and systemic inflammation [94,158]. TNF-α binds two types of soluble receptors (sTNFR1 and sTNFR2). While sTNFR1 is found in nearly all cells, sTNFR2 is present in the immune cells and the heart [159]. TNF-α is also involved in the alteration of vascular morphology and function. It may stimulate the synthesis of adhesion molecules (VCAM and ICAM) and MCP-1, in endothelial cells and VSMCs [158,159], induce the synthesis of reactive oxygen spices, and enhance systemic inflammation [94,159]. It can also increase the stiffness of the vascular endothelial cells [160] and stimulate collagen deposition by fibroblasts [161]. All these mechanisms may contribute to endothelial dysfunction and vascular remodeling.

IL-6 is produced by adipocytes, endothelial cells, VSMCs, and macrophages [157,158]. In obesity, the main source of IL-6 is the visceral adipose tissue [162]. Increased circulating cholesterol can stimulate IL-6 expression in perivascular adipose tissue, which may subsequently contribute to vascular wall alterations and arterial stiffness [163].

The vascular effects of IL-6 are mediated by multiple mechanisms. Depending on the signaling pathway activated—classic or trans-signaling—Il-6 can produce different effects. IL-6 exerts its action by binding to its surface receptors, IL-6R. Soluble forms of IL-6R combined to IL-6 can induce glycoprotein 130 (gp130) dimerization in cells that do not express IL-6R, rendering them responsive to IL-6 through a process called trans-signaling [164]. In endothelial cells, IL-6 induces the up-regulation of the adhesion molecules (ICAM-1, VCAM-1 and E-selectin)—which are proatherogenic—via a trans-signaling mechanism [158]. IL-6 can also contribute to the differentiation of monocytes to macrophages, stimulates MMP-2 and MMP-9 production [165], and increases CRP hepatic synthesis, enhancing inflammatory reactions [26].

Obesity and metabolic syndrome are accompanied by persistently increased IL-6 and TNF-α levels which may contribute to insulin resistance and deleterious vascular effects [159,162]. TNF-α and IL-6 are also involved in inflammation-induced arterial stiffness [166].

In patients with acute ischemic stroke and metabolic syndrome, measures of arterial stiffness (carotid–femoral PWV and aortic augmentation index) and inflammatory cytokines (TNF-α and IL-6), along with CRP, were increased compared to patients with acute ischemic stroke without metabolic syndrome. TNF-α, IL-6, and CPR more strongly correlated with arterial stiffness parameters in patients with ischemic stroke and metabolic syndrome compared to those with acute ischemic stroke but without metabolic syndrome [167]. Increased systemic inflammation status from metabolic syndrome together with insulin resistance might have explained the increased risk of vascular disease, but the precise mechanisms are not yet clarified.

Soluble TNF-α receptors—sTNFR1 and sTNFR2—were positively associated with brachial–ankle PWV in patients with coronary artery disease undergoing invasive coronary angiography, independently of blood pressure and aging [168]. A positive and independent correlation between sTNFR2 and brachial–ankle PWV was reported in diabetic non-obese Japanese patients [169], and between sTNFR1 and carotid–femoral PWV in healthy women [170]. In these two last mentioned studies, arterial stiffness parameters were correlated with TNF-α receptors but not with TNF-α itself, suggesting that circulating soluble TNF-α receptors can be better markers of vascular stiffness [169,170].

The implication of TNF-α in vascular stiffness is also supported by the fact that anti-TNF-α agents used to treat inflammatory arthritis patients with increased arterial stiffness had favorable effects on stiffness parameters [171].

IL-6 correlated with carotid distensibility in uncomplicated type 2 diabetes mellitus, mediating the relation between abdominal fat and carotid distensibility [172]. IL-6 correlated with arterial stiffness in a healthy Greek population [173], and also in hypertensive never-treated subjects [174]. IL-6 and its soluble receptors, sIL-6R and soluble gp130 receptors were positively associated with serological markers of endothelial function (E-selectin, I-CAM-1, V-CAM-1) and inversely with pulse wave propagation time, a marker of arterial stiffness in patients with metabolic syndrome [175]. This article indicated the involvement of the trans-signaling process in vascular stiffness.

In healthy women, the soluble receptor of IL-6 but not IL-6, inversely correlated with flow-mediated dilation. Like TNF-α receptors, soluble IL-6 receptors seem to be better indicators of early vascular changes than the interleukin itself [170]. Moreover, it has been reported that inhibition of IL-6 with tocilizumab, an antibody which binds to both soluble and membrane IL-6 receptors, improving endothelial function and arterial stiffness in patients with rheumatoid arthritis [176]. However, this effect has not been proven in all studies [171].

The IL-6–174-G/C promoter polymorphism, previously associated with inflammation, has been investigated in relation to arterial stiffness in a large population-based sample from the Rotterdam study. The results indicated a significant correlation between the C-allele of the polymorphism and increased carotid–femoral PWV. A tendency, even though statistically insignificant, of association between IL-6 and increased PWV, was also reported, but the number of IL-6 samples was small [177].

### 3.10. Serum Amyloid A(SAA)

SAA is an acute phase protein produced by the liver, but it can also be synthesized by other cells including macrophages and adipocytes [178]. Its hepatic synthesis is induced by inflammatory cytokines such as IL-1, IL-6 and TNF α. SAA is an apolipoprotein of high-density lipoprotein cholesterol (HDLc) replacing apolipoprotein A-I during the acute inflammatory response and transforming HDLc into a proatherogenic and proinflammatory molecule. An oxidative and inflammatory environment may favor the formation of the SAA-low-density lipoprotein (SAA-LDL) complex, which is an oxidized LDL involved in endothelial dysfunction and atherosclerosis development [179]. Moreover, SAA-LDL may be involved in various mechanisms that interfere with vascular structure and function. It has been shown that SAA-LDL can favor the transformation of the quiescent and contractile VSMC form into a synthetic, proliferative form [180], and stimulate vascular proteoglycan synthesis [181] and the expression of phospholipase A2 [182]. SAA also induces the expression of adhesion molecules (ICAM-1, VCAM-1) and matrix metalloproteinases (MMP-2 and MMP-9) by the activation of the NF-κB-dependent pathway [183]. Thus, it can be speculated that SAA-LDL, may reflect systemic inflammation and be a link between inflammation and vascular disease.

SAA is increased in obesity and metabolic syndrome [184] and it could be a link or even a mediator between obesity and cardiovascular diseases [185]. Increased levels of SAA have been shown to predict cardiovascular events [185,186,187].

SAA has also been studied in relation to preclinical vascular disease. Increased SAA levels were positively correlated with CAVI in Japanese adults, which were non-medicated and free of cardiovascular disease [188], as well as in type 2 diabetic patients [189]. However, in a young population from the Cardiovascular Risk in Young Finns Study, SAA was not independently associated with carotid distensibility and IMT. Nevertheless, SAA correlated with metabolic risk markers which may serve as mediators in the interaction between SAA and vascular pathology [190].

### 3.11. Monocyte Chemoattractant Protein-1 (MCP-1)

MCP-1 is a chemokine produced by various cells, particularly monocytes/macrophages and endothelial cells, and has an important role in local inflammation. MCP-1 binds on its receptor chemokine (C-C motif) ligand 2 (CCL2), expressed in monocytes, and attracts them to the site of inflammation. Once recruited, monocytes enhance inflammation through the secretion of inflammatory cytokines such as IL-6 and TNF-α in endothelial cells [158,191]. MCP-1 expression is increased in adipose tissue, proportionally to adiposity, and contributes to macrophage infiltration and insulin resistance in obesity [192]. Inflammatory cytokines, oxidized LDL, and other pro-inflammatory molecules may stimulate MCP-1 synthesis [158,193].

MCP-1 expression is mainly localized to the thickened intima and it promotes VSMCs proliferation and migration, contributing to vascular remodeling [193]. MCP-1 is also involved in the initiation of vascular wall fibrosis, increasing the activation of TGF-β1 [194]. Moreover, TGF-β1 contributes to VSMCs stiffening [193].

All these mechanisms indicate that MCP-1 is involved in vascular inflammation and remodeling which may induce arterial stiffness. However, the results of clinical studies are conflicting. In a large sample of young healthy subjects, from the African-PREDICT Study, MCP-1 did not correlate with arterial stiffness. Its values were higher in young black Africans compared to white subjects, and positively associated with carotid IMT in black women, suggesting that MCP-1 may contribute to early development of vascular alteration in this population, which is known to be at increased cardiovascular risk [195]. In children with type 1 diabetes mellitus, MCP-1 was increased but it did not correlate with arterial stiffness. It has been suggested that increased MCP-1 may activate endothelial pathological process even though arterial stiffness is not yet developed [196]. However, in diabetic and non-diabetic predialysis chronic kidney disease 3–5 patients, MCP-1 was increased in diabetic compared to non-diabetic chronic kidney disease patients. Along with mean arterial pressure and serum glycated hemoglobin, MCP-1 independently associated with increased CAVI [197].

## 4. Perivascular Adipose Tissue and Vascular Remodeling and Stiffness

Perivascular adipose tissue is the adipose fat which surrounds large arteries (aorta), medium-size (mesenteric arteries) and small arteries (resistance arteries). Epicardial fat plays the role of the perivascular adipose tissue for coronary arteries. In lean subjects, perivascular adipose tissue is a source of adipokines with favorable effects. Among them, the most important is adiponectin which has vasodilatory effects on small arteries, stimulates local endothelial NO synthesis, reduces inflammation-suppressing adipocytes’ secretion of CRP, IL-6, and TNF-α, inhibits VSMC proliferation, and prevents monocyte adhesion to aortic endothelial cells [198]. In obesity, increased perivascular adipose tissue has been associated with arterial stiffness [198,199]. The influence of perivascular fat on arterial stiffness seems to be independent of the total body fat mass [198]. Hypertrophic adipocytes of perivascular tissue produce inflammatory cytokines, (i.e., IL-6, TNF-α, MCP-1) which stimulate the inflammation and invasion of immune cells into the vessel wall. IL-6 may enhance collagen type 1 synthesis and the accumulation of advanced glycation end products in the vascular wall. Leptin and visfatin were associated with VSMC phenotypic switching to a synthetic phenotype that further plays an essential role in vascular remodeling and the deposition of calcium phosphate in the vascular wall [199,200]. Inflammation and oxidative stress in obese perivascular adipose tissue are involved in endothelial disfunction and may alter vascular tone, favoring vasoconstriction [201,202]. The activation of perivascular adipose fat RAAS, which enhances oxidative stress and inflammation in vascular tissue, is another important contributor to arterial stiffness [202].

## 5. Conclusions

Adipokines are active molecules synthetized by the adipose tissue, with various metabolic and vascular functions. Obesity, characterized by persistent low-grade inflammation, is accompanied by a disturbed balance of secretion of proinflammatory/inflammatory adipokines secretion. Increased proinflammatory adipokines are important mediators of obesity-associated pathologies, including cardiovascular diseases. Various mechanisms can explain the implication of adipokines in arterial stiffness through the activation of vascular inflammation and oxidative stress, the stimulation of vascular fibrosis and calcification, and the precise roles of adipokines are not yet clarified. From a clinical viewpoint, the use of different measures of arterial stiffness and the various pathologies associated in obese patients, make difficult the comparison between studies.

In addition to circulating adipokines, recent research has highlighted the role of perivascular adipose tissue adipokines, in the development of arterial stiffness in obesity. Nevertheless, the confirmation of a cause–effect relationship between adipokines and vascular stiffness needs futures prospective large studies.

## Data Availability

Not applicable.
